# Frontotemporal dementia and amyotrophic lateral sclerosis-associated disease protein TDP-43 promotes dendritic branching

**DOI:** 10.1186/1756-6606-2-30

**Published:** 2009-09-25

**Authors:** Yubing Lu, Jacob Ferris, Fen-Biao Gao

**Affiliations:** 1 Gladstone Institute of Neurological Disease, and Department of Neurology, University of California, San Francisco, CA 94158 USA

## Abstract

**Background:**

TDP-43 is an evolutionarily conserved RNA-binding protein implicated in the pathogenesis of frontotemporal dementia (FTD), sporadic and familial amyotrophic lateral sclerosis (ALS), and possibly other neurodegenerative diseases. In diseased neurons, TDP-43 is depleted in the nucleus, suggesting a loss-of-function pathogenic mechanism. However, the normal function of TDP-43 in postmitotic neurons is largely unknown.

**Results:**

Here we demonstrate that overexpression of *Drosophila *TDP-43 (dTDP-43) in vivo significantly increases dendritic branching of sensory neurons in *Drosophila *larvae. Loss of dTDP-43 function, either in a genetic null mutant or through RNAi knockdown, decreased dendritic branching. Further genetic analysis demonstrated a cell-autonomous role for dTDP-43 in dendrite formation. Moreover, human TDP-43 (hTDP-43) promoted dendritic branching in *Drosophila *neurons, and this function was attenuated by mutations associated with ALS.

**Conclusion:**

These findings reveal an essential role for TDP-43 in dendritic structural integrity, supporting the notion that loss of normal TDP-43 function in diseased neurons may compromise neuronal connectivity before neuronal cell loss in FTD and ALS.

## Background

Frontotemporal lobar degeneration (FTLD) is a devastating age-dependent neurodegenerative condition primarily associated with impairments in cognition and social behaviors, as well as personality changes and other clinical abnormalities [1]. Frontotemporal dementia (FTD), a major clinical syndrome of FTLD, is now recognized as the most common form of early-onset age-dependent dementia before the age of 60 [2]. Increasing clinical, pathological, and molecular evidence indicates that FTD and amyotrophic lateral sclerosis (ALS) are closely related conditions [3].

In addition to pathogenic mutations in the microtubule binding protein tau [4,5], rare mutations in other genes also cause FTD, such as those encoding valosin-containing protein, an AAA-type ATPase associated primarily with the endoplasmic reticulum [6], and CHMP2B, a component of the endosomal sorting complex required for transport III [7]. Mutations in progranulin located on chromosome 17 also cause FTD in some patients without tau pathology [8,9]. Progranulin is a secreted molecule, and many pathogenic mutations lead to progranulin haploinsufficiency. The pathogenic mechanism of FTD caused by progranulin deficiency is not known, but one of the pathological hallmarks is tau-negative and ubiquitin-positive neuronal inclusions that contain TDP-43 and its fragments [10-13]. Genetic mutations in *TDP-43 *are responsible for some sporadic and familial amyotrophic lateral sclerosis (ALS) [14-16], further reinforcing the notion that FTD and ALS are closely related conditions, referred to as TDP-43 proteinopathies [17]. In healthy cells, TDP-43 mostly resides in the nucleus. In diseased neurons, however, TDP-43 is excluded from the nucleus and aggregates in the cytosol [10]. Moreover, in axotomized motor neurons, TDP-43 expression is dramatically increased and becomes prominently localized in the cytosol [18]. These findings raise the possibility that loss of the normal function of TDP-43, especially in the nucleus, contributes to the initiation or progression of disease.

TDP-43 is a ubiquitously expressed RNA-binding protein that contains two RNA recognition motifs, a glycine-rich region, a nuclear localization signal, and a nuclear export signal [19]. It is not known which aspects of cellular physiology are regulated by TDP-43. TDP-43 is primarily localized in the nucleus at the active sites of transcription and cotranscriptional splicing in mammalian neurons [20]. Indeed, limited experimental evidence indicates that TDP-43 is involved in transcription [21] and splicing [22,23] and possibly in mRNA transport and local translation as well [24]. TDP-43 and many other proteins form a large complex with Drosha [25], but its possible involvement in the microRNA pathway remains to be further explored. To understand how TDP-43 contributes to the pathogenesis of FTD and ALS, it is essential to dissect its normal functions in postmitotic neurons.

TDP-43 is highly conserved at the amino acid level from flies to humans, suggesting an evolutionarily conserved function [19,23,26]. To investigate the normal roles of TDP-43 in postmitotic neurons, we used dendrites of sensory neurons in the *Drosophila *peripheral nervous system (PNS) as our assay system and performed both gain- and loss-of-function genetic studies. We also examined the functional significance of some genetic mutations in TDP-43 that are associated with ALS. Our findings support the notion that a TDP-43 loss-of-function mechanism may contribute to the pathogenesis of FTD and ALS.

## Methods

### Fly strains and genetics

All flies were raised on standard food medium and kept at 25°C. *dTDP-43 *RNAi lines 38377 and 38379 were obtained from the Vienna *Drosophila *RNAi Center (VDRC). The *dTDP-43*^*Q*367*X *^mutant allele was identified from the Seattle *Drosophila *TILLING Project (Fly-TILL, Fred Hutchinson Cancer Research Center) using specific tilling primers (Additional file 1). *dTDP-43*^*Q*367*X*^/*CyO, GFP *flies were crossed with *Pin/CyO, GFP; Gal4*^221^, *UAS-mCD8-GFP *to establish the stock *dTDP-43*^*Q*367*X*^/*CyO, GFP; Gal4*^221^, *UAS-mCD8-GFP/+*. The *Gal4*^221 ^driver was used to label ddaE and ddaF neurons with mCD8-GFP and drive the expression of transgenes [27]. To visualize dendritic phenotypes of ddaE and ddaF neurons in third instar larvae, we crossed *dTDP-43*^*Q*367*X*^/*CyO, GFP; Gal4*^221^, *UAS-mCD8-GFP/+ *flies with *dTDP-43*^*Q*367*X*^/*CyO*, *GFP *or *w*^1118 ^flies to generate *dTDP-43*^*Q*367*X*^/*dTDP-43*^*Q*367*X*^*; Gal4*^221^, *UAS-mCD8-GFP/+ *or *dTDP-43*^*Q*367*X*^/+; *Gal4*^221^, UAS-*mCD8-GFP/+ *third instar larvae. For RNAi expression, *dTDP-43*^*Q*367*X*^/*CyO, GFP; Gal4*^221^, *UAS-mCD8-GFP/+ *flies were crossed with UAS-*dTDP-43 *RNAi lines (VDRC 38377 and 38379) to generate *dTDP-43*^*Q*367*X*^/*+; Gal4*^221^, UAS-*mCD8-GFP/38377, and dTDP-43*^*Q*367*X*^/*38379; Gal4*^221^, UAS-*mCD8-GFP/+ *third instar larvae for phenotypic analysis. For transgene overexpression, *Gal4*^221^, *UAS-mCD8-GFP *flies were crossed with *UAS-dTDP-43,, UAS-hTDP-43, UAS-hTDP-43-M337V*, or *UAS-hTDP-43-Q331K *transgenic lines. In the above experiments, *Gal4*^221^, *UAS-mCD8-GFP/+ *third larvae served as the control.

### Generation of transgenic *Drosophila *lines

Full-length *hTDP-43 *cDNA was cloned from HEK293 cells (provided by Dr. J.-A. Lee). To generate *UAS-hTDP-43*, *UAS-hTDP-43-M337V, UAS-hTDP-43-Q331K, and UAS-hTDP-43-*C-terminal fragment (amino acids 209-414) constructs, the corresponding primers (Additional file 1) were used to amplify DNA fragments, which were then cloned into the pUAST vector. To generate *UAS-dTDP-43 *constructs, the full-length dTDP-43 coding sequence was amplified from the cDNA plasmid GH09868 (*Drosophila *Genomics Resource Center) and cloned into the pUAST vector. These constructs were confirmed by sequencing and microinjected into wild-type (*w*^1118^) embryos to generate transgenic lines.

### Antibody production and western blot

Anti-dTDP-43 polyclonal antibody was generated by immunizing rabbits with a peptide fragment spanning amino acids 179-192 (Thermo Fisher Scientific). For protein expression analysis, adult flies were frozen in ethanol with dry ice and vortexed to remove heads. Approximately 30 heads from each genotype were homogenized in 50 μl of lysis buffer (0.137 M NaCl, 20 mM Tris, pH 8.0, 10% glycerol, 1% NP-40, 0.1% SDS, 0.1% sodium deoxycholate, 1 mM DTT, Pierce protease inhibitors and phosphatase inhibitors). Homogenate was heated at 65°C for 10 min and centrifuged at 4°C for 10 min at 13,000 rpm. Protein concentrations were determined using Bradford Assay (Bio-Rad).

Supernatant containing 10 μg of protein was separated on a 10% acrylamide SDS gel and transferred to a PVDF membrane (Bio-Rad) in a wet transfer system at 4°C for 60 min at 100 V. The membrane was incubated in blocking solution containing 5% milk in TBST (25 mM Tris-HCl, 137 mM NaCl, 3 mM KCl, pH 7.4, and 0.1% Tween-20) at 4°C overnight, with dTDP-43 antibody (1:1000 in blocking solution) at room temperature for 3 h, and finally with anti-rabbit HRP-conjugated secondary antibody (Jackson Immunoresearch; 1:10,000) for 1 h. The signal was visualized with chemiluminescent substrate (Supersignal West Pico, Pierce). For other western blot analyses, the primary antibodies were hTDP-43 antibody (1:1000; 10782-2-AP, Proteintech), and β-actin antibody (1:1500; Cell Signaling).

### Quantitative RT-PCR (qRT-PCR) analysis

Total RNAs were extracted from adult heads with Trizol (Invitrogen) and used as templates to generate cDNAs with TaqMan reverse transcription reagent (Applied Biosystems). cDNAs were used as templates for qRT-PCR in a final volume of 25 μl. A standard curve was run in each PCR reaction. Individual values were normalized to the value of the gene encoding the ribosomal protein RP-49. Two pairs of primers were designed to detect dTDP-43 transcripts (Additional file 2). All reactions were performed three times. Relative mRNA expression was calculated using the standard curve method and the delta-delta Ct method.

### Mosaic analysis with a repressible cell marker (MARCM)

MARCM analysis of sensory neurons in the *Drosophila *PNS was performed as described [28]. First, the *dTDP-43*^*Q*367*X *^allele was recombined onto the chromosome containing *FRT*^*G*13^. *FRT*^*G*13^, *dTDP-43*^*Q*367*X*^/*CyO *or *FRT*^*G*13^/*CyO *male flies were crossed with *Gal4*^*C*155^, *UAS-mCD8-GFP, hs-FLP1; FRT*^*G*13^, *Gal80/CyO *virgin females to generate *Gal4*^*C*155^, *UAS-mCD8-GFP, hs-FLP1/+; FRT*^*G*13^, *Gal80/FRT*^*G*13^, *dTDP-43*^*Q*367*X *^and *Gal4*^*C*155^, *UAS-mCD8-GFP, hs-FLP1/+; FRT*^*G*13^, *Gal80/FRT*^*G*13 ^embryos, respectively. Embryos from these crosses were collected on grape agar plates for 3 h in a 25°C incubator. The embryos were aged for 3 h and heat-shocked in a 37°C water bath for 40 min to induce mitotic recombination. The embryos were then kept in a moisture chamber at 25°C for 3-4 days. Third instar larvae were collected, and larvae that contained a single mCD8::GFP-labeled dorsal cluster PNS neuron were selected under a Nikon fluorescence dissection microscope. Images of the dendritic morphology of single DA neurons were recorded with a confocal microscope (Nikon, D-Eclipse C1). The significance of differences in dendritic branching complexity was determined with Student's *t *test.

### Quantitative Analysis of dendritic ends of sensory neurons

The dendritic morphology of GFP-labeled dorsal sensory neurons was recorded with a confocal microscope (Nikon, D-Eclipse C1), and dendritic branches of ddaE or ddaF neurons in the A3 dorsal cluster were counted as described [28]. Briefly, dendritic ends of DA neuron images were identified visually and highlighted with dots, which were counted with Adobe Photoshop software. The data were analyzed by Student's *t *test.

## Results

### dTDP-43 promotes dendritic branching of sensory neurons in *Drosophila*

To examine neuronal functions of dTDP-43, we generated multiple UAS-*dTDP-43 *transgenic fly lines with insertion sites on different chromosomes. Because dendritic branching patterns are critically important for neuronal function and connectivity and dendritic defects are associated with many neurological disorders [29,30], we focused our functional analysis of TDP-43 on dendrites. To do so, we used the sensory neurons in *Drosophila *larvae as our assay system, which has been useful in uncovering molecular mechanisms of dendritic morphogenesis [31]. To drive transgene expression, we used the neuronal subtype-specific driver Gal4^221^, which targets gene expression in two sensory neurons only in each hemisegment [27].

**Figure 1:**
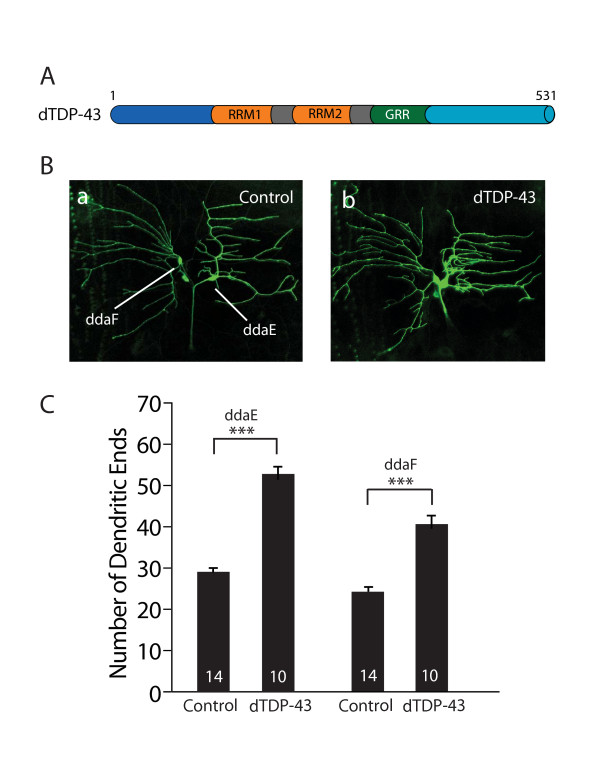
**dTDP-43 promotes dendritic branching in *Drosophila***. (A) Schematic representation of the domain structure of dTDP-43. RRM: RNA recognition motif. GRR: glycine-rich region. (B) Dendritic phenotypes of ddaE and ddaF sensory neurons in the A3 segment of third instar larvae that are either wildtype (a) or overexpress dTDP-43 with the *Gal4*^221 ^driver (b). (C) Quantification of dendritic ends of ddaE and ddaF neurons. The number of neurons examined is indicated in each column. Values are mean ± SEM. ***P < 0.001.

Ectopic expression of dTDP-43 markedly increased the number of small terminal dendritic branches (Figure 1B). For instance, the number of dendritic ends increased from 29.2 ± 1.0 (n = 14) to 51.2 ± 3.0 (n = 10) (*P *< 0.001) in ddaE neurons, and from 22.8 ± 1.0 (n = 14) to 39.9 ± 1.9 (n = 10) (*P *< 0.001) in ddaF neurons (Figure 1C). Interestingly, most of the small terminal dendritic branches caused by dTDP-43 expression were concentrated near the cell body of ddaE neurons (Figure 1B). This effect of dTDP-43 was confirmed in studies with other UAS-*dTDP-43 *transgenic fly lines with different insertion sites on the second or third chromosomes (data not shown).

### dTDP-43 is required for normal viability of adult flies

**Figure 2:**
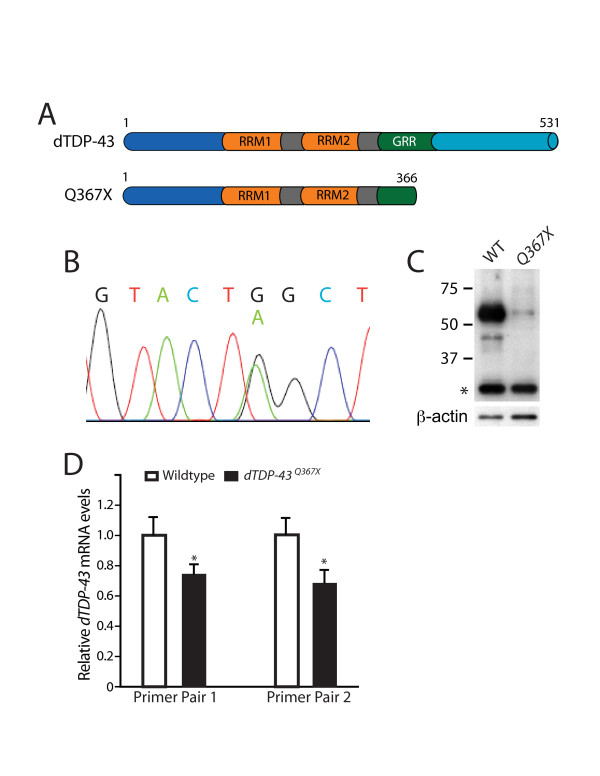
**Generation of a *dTDP-43 *null allele**. (A) Schematic representation of the truncated dTDP-43 protein caused by the single nonsense nucleotide mutation at amino acid 367. (B) Sequencing of the PCR fragment amplified from the genomic DNA of heterozygous flies confirmed the single nucleotide mutation from G to A. (C) Western blot analysis of protein lysates from the heads of wildtype (WT) or *dTDP-43*^*Q*367*X *^homozygous mutant adult flies confirmed the absence of full-length dTDP-43 in mutants. The asterisk indicates a nonspecific band recognized by this antibody. The rabbit polyclonal antibody was raised against the peptide fragment spanning amino acids 179-192 and did not recognize the putative truncated dTDP-43 in mutants, indicating instability of the mutant *dTDP-43 *mRNA or protein. (D) qRT-PCR analysis of *dTDP-43 *mRNA levels in wildtype and *dTDP-43*^*Q*367*X *^mutant flies. Two pairs of primers (Additional file 2) used for qRT-PCR yielded similar results. Values are mean ± SD. *P < 0.05.

Next, we generated *dTDP-43 *loss-of-function mutant flies. We used the service from the Seattle *Drosophila *TILLING Project (Fred Hutchinson Cancer Research Center) and obtained several fly lines in which point mutations were found in *dTDP-43*. One, a nonsense mutation with a single nucleotide change from G to A, introduced a stop codon at amino acid 367 (Figure 2A). This mutation was confirmed by sequencing PCR products amplified from the genomic DNA of *dTDP-43*^*Q*367*X *^heterozygous adult flies (Figure 2B). To confirm that this mutant allele is a protein-null allele, we raised a rabbit polyclonal antibody against the dTDP-43 peptide fragment spanning amino acids 179-192. On western blot, this antibody recognized a protein band with the predicted molecular weight of dTDP-43 in lysates from adult wildtype but not in *dTDP-43*^*Q*367*X *^mutant flies (Figure 2C). The intensity of this band was decreased by about half in *Df(2R)106*/+ heterozygous flies (data not shown). Since this antibody did not detect the putative truncated dTDP-43 protein in mutant flies (Figure 2C), it is likely that the truncated dTDP-43 protein is unstable, in addition to the reduced abundance of the *dTDP-43 *mRNA containing the premature stop codon (Figure 2D). Thus, *dTDP-43*^*Q*367*X *^is a protein null allele.

Homozygosity for *dTDP-43*^*Q*367*X *^was semi-lethal, with some mutant adult flies surviving to adulthood. For instance, the ratio of *dTDP-43*^*Q*367*X*^/*CyO *to *dTDP-43*^*Q*367*X*^/*dTDP-43*^*Q*367*X *^progenies of *dTDP-43*^*Q*367*X*^/*CyO *flies was about 4-5:1, instead of the expected 2:1 ratio for nonlethal mutations. This finding is consistent with a recent report that *dTDP-43 *deletion mutations in were semi-lethal as well [26]. Expression of UAS-*dTDP-43 *RNAi (38377 or 38379, VDRC) driven by *tubulin*-*Gal4 *resulted in a similar lethal phenotype. Overexpression of dTDP-43 with the pan-neuronal *Gal4*^*C*155 ^driver also led to a similar lethal phenotype, indicating that the proper level of dTDP-43 expression in the nervous system is required for normal viability. Indeed, ectopic expression of dTDP-43 in the eye caused a severe retinal degeneration phenotype (data not shown).

### Loss of dTDP-43 activity reduces dendritic branching

**Figure 3:**
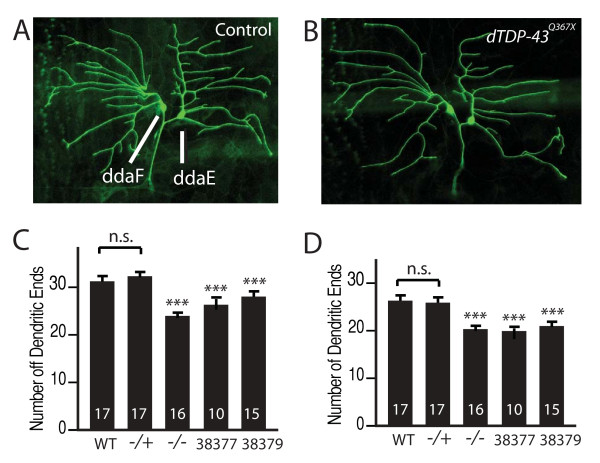
**Loss of dTDP-43 activity led to decreased dendritic branching**. (A) Dendritic branching pattern of ddaE and ddaF neurons in a wildtype larva. (B) ddaE and daF neurons in a *dTDP-43*^*Q*367*X *^homozygous mutant third instar larva. The neurons in A and B were labeled with mCD8-GFP under the control of *Gal4*^221^. (C) Quantification of dendritic ends of ddaE neurons. (D) Quantification of dendritic ends of ddaF neurons. The number of neurons examined is indicated in each column. Values are mean ± SEM ***P < 0.001. The effect of loss of dTDP-43 function on dendritic formation was confirmed by expressing two independent UAS-*dTDP-43 RNAi *constructs, 38377 and 38379, in ddaE and ddaF neurons in the *dTDP-43*^*Q*367*X*^/+ background.

To examine dendritic phenotypes caused by loss of dTDP-43 activity, we first expressed mCD8-GFP under the control of *Gal4*^221 ^in ddaE and ddaF neurons in wildtype or *dTDP-43*^*Q*367*X *^homozygous mutant third instar larvae. Compared with wildtype neurons (Figure 3A), loss of dTDP-43 function significantly decreased dendritic branching (Figure 3B). For instance, the number of dendritic ends of ddaE neurons in the A3 segment decreased from 31.4 ± 1.0 (n = 17) in wildtype larvae to 24.4 ± 0.5 (n = 16) in *dTDP-43*^*Q*367*X *^homozygous mutant larvae (*P *< 0.001) (Figure 3C). A similar decrease was observed in ddaF neurons (Figure 3D).

Since ectopic overexpression of dTDP-43 markedly increased dendritic branching, it would be difficult to interpret results obtained by using the UAS-Gal4 system to rescue the dendritic phenotype in *dTDP-43*^*Q*367*X *^mutants. Therefore, to further confirm that loss of dTDP-43 activity reduces dendritic branching, we used two independent *dTDP-43 *RNAi lines obtained from VDRC to knock down dTDP-43 expression in these neurons. Again, the number of dendritic ends was decreased in ddaE (Figure 3C) and ddaF neurons (Figure 3D). These findings demonstrate that dTDP-43 has an essential role in patterning dendritic formation in vivo.

### dTDP-43 has a cell-autonomous function in dendritic branching

**Figure 4:**
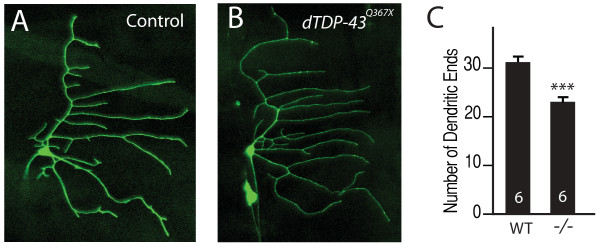
**MARCM analysis reveals a cell-autonomous role for dTDP-43 in dendritic branching**. (A) A wildtype ddaE neuron labeled with GFP by MARCM. (B) A *dTDP-43*^*Q*367*X *^mutant ddaE neuron labeled with GFP by MARCM. (C) Quantification of dendritic ends of ddaE neurons. The number of neurons examined is indicated in each column. Values are mean ± SEM. ***P < 0.001.

In the RNAi experiment described above, dTDP-43 activity was knocked down in only a small number of sensory neurons, suggesting that dTDP-43 functions in a cell-autonomous manner to control dendritic branching. To further confirm this possibility, we used the mosaic analysis with a repressible cell marker (MARCM) technique, which allows one to generate GFP-labeled single neurons containing a mutation of a gene of interest in a mostly wildtype *Drosophila *larva or adult [32]. Using this approach, we found that compared with wildtype ddaE neurons (Figure 4A), loss of dTDP-43 function specifically in ddaE neurons reduced dendritic branching (Figure 4B), consistent with the genetic analysis of these neurons in *dTDP-43*^*Q*367*X *^homozygous mutant larvae (Figure 3). *dTDP-43*^*Q*367*X *^mutant ddaE neurons in the A3 segment had fewer dendritic ends than wildtype neurons labeled by MARCM (23.3 ± 3.4 (n = 6) vs. 31.3 ± 4.9 (n = 6) (*P *< 0.001). This result indicates that dTDP-43 functions in a cell-autonomous manner to regulate dendritic branching.

### hTDP-43 promotes dendritic branching in fly neurons and ALS-associated disease mutations attenuates this activity

**Figure 5:**
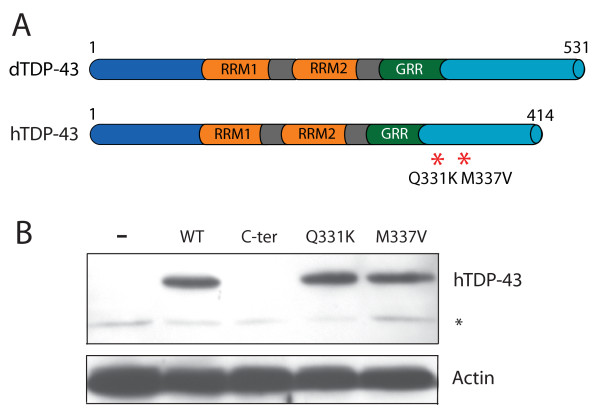
**Generation of transgenic flies that express wildtype and mutant hTDP-43**. (A) Schematic representations of dTDP-43 and hTDP-43 and the positions of ALS-associated point mutations. RRM: RNA recognition motif. GRR: glycine-rich region. (B) Western blot analysis of the levels of wildtype or mutant hTDP-43 expressed in the eye with the *GMR-Gal4 *driver. The dTDP-43 antibody did not recognize the hTDP-43 C-terminal fragment. The asterisk indicates a nonspecific band. Actin was used as the loading control.

TDP-43 is an evolutionarily conserved protein whose amino acid sequence is highly homologous in flies and humans. To examine whether human TDP-43 (hTDP-43) also promotes dendritic branching, we generated multiple lines of UAS-*hTDP-43 *transgenic flies (Figure 5A). The C-terminal fragment of hTDP-43 and several point mutations have been implicated in the pathogenesis of FTD and ALS [10,14-17], but the mechanisms are unclear. To address this issue, we also generated multiple transgenic fly lines to express these mutant hTDP-43 proteins (Figure 5A). The level of transgene expression can vary depending on the insertion site in the genome. We therefore performed western blot analysis and selected individual transgenic fly lines that, when crossed with the *GMR*-Gal4 line, expressed hTDP-43^WT ^and hTDP-43^Q331K ^or hTDP-43^M337V ^at comparable levels (Figure 5B).

**Figure 6:**
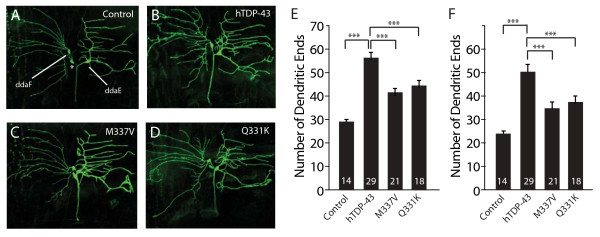
**hTDP-43 promotes dendritic branching in *Drosophila *neurons and disease-related mutations in *hTDP-43 *reduce this activity**. (A-D) Images of ddaE and ddaF neurons that express mCD8-GFP (A), wildtype hTDP-43 (B), hTDP-43 with the Q331K mutation (C), and hTDP-43 with the M337V mutation (D). The asterisk indicates another sensory neuron in the cluster that occasionally was weakly labeled with GFP expressed with the *Gal4*^221 ^driver. (E) Quantification of dendritic ends of ddaE neurons. (F) Quantification of dendritic ends of ddaF neurons. The number of neurons examined is indicated in each column. Values are mean ± SEM. ***P < 0.001.

Ectopic expression of hTDP-43 in *Drosophila *neurons also promoted dendritic branching (Figure 6). The number of dendritic ends for ddaE neurons was increased from 29.5 ± 0.8 (n = 14) (Figure 6A) to 55 ± 1.6 (n = 29) (*P *< 0.001) (Figure 6B, E). A similar effect was observed for ddaF neurons (Figure 6B, F). The ectopic expression of the hTDP-43 C-terminal fragment did not affect dendrite branching (data not shown). As with dTDP-43, ectopic expression of hTDP-43 increased in dendritic ends mostly near the cell body (Figure 6B). These findings suggest that hTDP-43 and dTDP-43 are functionally conserved and likely exert their effects on dendritic branching through the same genetic pathways.

Both of those ALS-associated mutant proteins promoted dendritic branching to a much lesser extent than wildtype hTDP-43 (Figure 6C). ddaE neurons expressing hTDP-43^WT ^had 55 ± 1.6 (n = 29) dendritic ends but the neurons expressing hTDP-43^M337V ^or hTDP-43^Q331K ^had only 41.1 ± 1.5 (n = 18) or 44.3 ± 2.0 (n = 21) dendritic ends, respectively (*P *< 0.001) (Figure 6E). A similar effect was observed for ddaF neurons (Figure 6F). Thus, ALS-associated mutations in hTDP-43 attenuated its dendrite-promoting activity, raising the possibility that loss-of-function mechanism as a contributing factor to the disorder.

## Discussion

The pathological role of TDP-43 was first recognized by its presence in ubiquitin-positive but tau-negative inclusions in diseased neurons of FTD and ALS patients [10,11]. TDP-43 pathology has two characteristic features. First, TDP-43 is proteolytically processed, and phosphorylated C-terminal fragments of approximately 20-25 kDa are present in the insoluble inclusions [10]. Indeed, ectopic expression of these fragments in cultured cells results in aggregation [33]. Second, TDP-43 is transported from the nucleus, where it predominantly resides in healthy cells. These findings suggest that TDP-43 may contribute to neurodegeneration through both a toxic gain-of-function mechanism and a loss-of-function mechanism. These possibilities are not mutually exclusive. However, the precise roles of TDP-43 in postmitotic neurons remain largely unknown.

Since TDP-43 is highly conserved at the amino acid sequence level from flies to humans, *Drosophila *offers a powerful model system to examine the endogenous functions of TDP-43. We obtained *dTDP-43 *null mutant flies and found that dTDP-43 is required for normal viability, consistent with a study published during the preparation of our manuscript [26]. *TDP-43 *knockout mice have not been reported yet. Considering the high degree of conservation between dTDP-43 and hTDP-43, it is possible that TDP-43 is also essential for normal development in mammals. At the cellular level, multiple lines of evidence from our current study indicate that TDP-43 promotes dendritic branching in postmitotic neurons. This conclusion was based on overexpression studies, RNAi knockdown, and genetic analysis of a *dTDP-43 *null allele. TDP-43 seems to also regulate axonal structures at the *Drosophila *neuromuscular junction (NMJ) [26]. These findings indicate an essential role for TDP-43 in neuronal structural integrity.

In many neurodegenerative diseases, defects in synaptic connections are probably one of the earliest alterations preceding neurodegeneration [34]. Recent imaging studies in human brains suggest that specific functional connectivity networks are compromised in specific neurodegenerative conditions [35]. It is conceivable that loss of the normal nuclear function of TDP-43 in specific vulnerable neurons reduces dendritic complexity, which in turn leads to compromised neuronal connectivity in that specific neuronal circuitry. Thus, exclusion of TDP-43 from the nucleus through unknown pathways in diseased neurons may represent a loss-of-function mechanism that may contribute to the pathogenesis of FTD and ALS.

*Drosophila *is also an excellent model system for studying human disease proteins. We found that hTDP-43 also promotes dendritic branching in *Drosophila *neurons, indicating a functional conservation. More importantly, two point mutations associated with ALS attenuated the dendrite-promoting activity of hTDP-43. Both are located in a C-terminal region that mediates protein-protein interactions [36]. Thus, these mutations may compromise the normal functions of TDP-43 in neurons. It was reported that these missense mutations might also enhance the formation of TDP-43 aggregates [37]. Thus, multiple pathogenic mechanisms may work in concert to promote disease initiation and/or progression.

## Conclusion

Overexpression of dTDP-43 or hTDP-43 in vivo significantly increased dendritic branching in a *Drosophila *assay system. RNAi knockdown and genetic analysis of a *dTDP-43 *null allele revealed a cell-autonomous role for dTDP-43 in promoting dendrite formation. Mutations associated with some forms of ALS reduced the dendrite-promoting activity of hTDP-43, suggesting a loss-of-function pathogenic mechanism. These findings support the notion that loss of normal TDP-43 function may contribute to the pathogenesis of FTD and ALS. The fly model reported here and newly generated relevant reagents will facilitate studies to further elucidate the underlying molecular mechanisms.

## Competing interests

The authors declare that they have no competing interests.

## Authors' contributions

YL and FBG designed the experiments, and YL and JF performed the experiments. YL, JF, and FBG analyzed the data, and wrote the paper. All authors read and approved the final version of the manuscript.

## Supplementary Material

Additional file 1 Primers for different transgenic constructs and sequencing primers. Click here for file

Additional file 2 Primers for qRT-PCR analysis of *dTDP-43 *mRNA levels. Click here for file
