# Supplementary material for: Frontotemporal dementia and amyotrophic lateral sclerosis-associated disease protein TDP-43 promotes dendritic branching
Source: Mol Brain. 2009 Sep 25;2:30. doi: 10.1186/1756-6606-2-30 (PMC2762964; doi:10.1186/1756-6606-2-30)
Supplement: Additional file 1 — Primers for different transgenic constructs and sequencing primers. [file 1756-6606-2-30-S1.DOC]

**Additional file 1. Primers for different transgenic constructs and sequencing primers.**

| *UAS-hTDP-43* | T-UP: 5’-CGCTCGAGATGTCTGAATATATTC-3’  T-DOWN: 5’-GCTCTAGACTACATTCCCCAGCCA-3’ |
| --- | --- |
| *UAS-hTDP-43-C Terminal fragment* | TC-UP: 5’-CGCTCGAGATGGTCTTCATCCCCA-3’  TC-DOWN: 5’-GCTCTAGACTACATTCCCCAGCCA-3’ |
| *UAS-hTDP-43-M337V* | TCM1-REV: 5’-CATGCCCACCATACCCCA-3’  TCM1-FOR: 5’-TGGGGTATGGTGGGCATG-3’  T-UP: 5’-CGCTCGAGATGTCTGAATATATTC-3’  T-DOWN: 5’-GCTCTAGACTACATTCCCCAGCCA-3’ |
| *UAS-hTDP-43-Q331K* | TCM2-REV: 5’-ACTGCTCTTTAGTGCTGC-3’  TCM2-FOR: 5’-GCAGCACTAAAGAGCAGT-3’  T-UP: 5’-CGCTCGAGATGTCTGAATATATTC-3’  T-DOWN: 5’-GCTCTAGACTACATTCCCCAGCCA-3’ |
| *UAS-dTDP-43* | UT-1: 5’-CGCTCGAGATGGATTTCGTTCAAGTG-3’  UT-2: 5’-GCTCTAGATTAAAGAAAGTTTGACTTC-3’ |
| Sequencing primer UAST-5737 | 5’-CCAGCAACCAAGTAAATCAACTGC-3’ |
| Sequencing primer UAST-5432 | 5’-CCCATTCATCAGTTCCATAGGTTG-3’ |
| Tilling primer-FOR | 5’-AGAACAAGCGCAAGAGCGACGATAACT-3’ |
| Tilling primer-REV | 5’-GGGATCCAGGTTTCTTAGGGGATGGTT-3’ |
| hTDP-43 point mutation sequencing primer-FOR | 5’-GTGCGCTTCGGCTCTTACG-3’ |
| hTDP-43 point mutation sequencing primer-REV | 5’-TCCAGGTGGCCACGGTTCATCC-3’ |
