# Supplementary material for: Frontotemporal dementia and amyotrophic lateral sclerosis-associated disease protein TDP-43 promotes dendritic branching
Source: Mol Brain. 2009 Sep 25;2:30. doi: 10.1186/1756-6606-2-30 (PMC2762964; doi:10.1186/1756-6606-2-30)
Supplement: Additional file 2 — Primers for qRT-PCR analysis of dTDP-43 mRNA levels. [file 1756-6606-2-30-S2.DOC]

| *dTDP-43* mRNA  coding region | **Forward primer** | **Reverse primer** |
| --- | --- | --- |
| **Pair 1: 194-289** | **GATTTTGCCTGGGTGTGAAT** | **TTCTGACTGCGTATGGCAAG** |
| **Pair 2: 1247-1320** | **AGGGCGGCAATTTTCTATCT** | **CTTCCGTCCACCAAAGTTGT** |

****Additional file 2. Primers for qRT-PCR analysis of *dTDP-43* mRNA levels.****
